# Food safety knowledge, attitude and self-reported practice of secondary school students in Beijing, China: A cross-sectional study

**DOI:** 10.1371/journal.pone.0187208

**Published:** 2017-11-02

**Authors:** Yinchu Cheng, Yang Zhang, Jun Ma, Siyan Zhan

**Affiliations:** 1 Department of Epidemiology and Biostatistics, Peking University School of Public Health, Beijing, China; 2 Institute of Child and Adolescent Health of Peking University, Beijing, China; University of Campinas, BRAZIL

## Abstract

In China, food safety problems have occurred frequently in the past ten years, causing great concern for the public. Adolescents, with higher exposure to problematic food, represent a unique target for interventions aimed at risk reduction. To understand their food safety knowledge, attitude and practice, a cross-sectional survey using paper questionnaire was carried out among 4,220 students (median age of 14 years, 50.3% females) from nine secondary schools in 3 districts of Beijing. The findings showed that the majority of respondents (42.0%) had a high knowledge level. Significant association was found between food safety knowledge score (median = 11, IQR:9–13) and demographic characteristics of region, school type, residence type, habit of smoking and alcohol use, academic record and parents’ education background. In terms of attitude and practice, only 17% of those surveyed regarded China’s food safety situation as good, 53.6% regarded it as worrying; almost all students (96.5%) did pay attention to food safety; 95.3% of the students had worried about the safety of the food provided by the small restaurants and street peddlers, but in reality, as many as 69.4% often or occasionally bought food from peddlers around their campuses and nearly half continued the consumption of such food in spite of worrying about its safety problems. Overall, the food safety knowledge among Beijing secondary school students was relatively good. They did not show much confidence in the country’s food safety situation, but many aware of the safety issues did not bother to change or take action. The study suggests that more systematic and targeted education on food safety is necessary for this age group.

## Introduction

Unsafe food poses global health threats, endangering everyone[1]. Food can be poisoned at any point of production and distribution. Major sources of food poisoning include pathogenic microorganisms, toxic animals and plants, chemical contamination, illegal additives and toxic industrial waste[2]. According to the World Health Organization, foodborne and waterborne diarrheal diseases kill an estimated 2 million people annually, mostly children and particularly in developing countries[3], though serious foodborne disease outbreaks have happened on every continent in the past decade, partially due to inadequate food safety laws, weak regulatory systems, and lack of education for food handlers and consumers[4].

In China, food consumption behaviors, which are deeply affected by Chinese food culture, have profound implications on the country’s food safety issues[5]. Most notably, Chinese people tend to regard taste as the most valued element of food and sometimes even neglect safety concerns of the food simply because they find it delicious[6]. A recent study in Beijing shows that about 75% of students once a week eat food served by unlicensed peddlers, which is usually very tasteful but may have safety problems[7]. Another fact is many Chinese prefer to cook with copious amount edible oil and thus produce a lot of waste oil which might be illegally collected and re-sold to restaurants as the so-called ‘gutter oil’[5,8]. Moreover, Chinese people has too wide a selection of food materials but not sufficient knowledge to deal with them. Some of them are more vulnerable to microorganisms during storing and transportation[2,6]. Besides cultural reasons, Chinese eating behaviors are also shaped by increasingly faster life style. People are more likely to eat outside their homes and thus more exposed to food problems in the public.

In recent years, China has seen several notorious problems concerning public food safety[9,10]. For instance, the contamination of infant formula with melamine in 2008 affected around 300,000 infants and young children countrywide, six of whom died[3]. These problems, together with the improper use of food additives, agrochemicals, antibiotic and antiparasitic agents in animal agriculture, have caused great concern for the public[2,11], leading to a change in their attitude toward food safety. People started to lose trust in food industry and confidence in the regulatory system. According to the KAP (knowledge, attitude and practice) model, a sound eating behavior develops based on a positive attitude, and a positive attitude is derived from solid knowledge on nutrition and food safety[12]. However, it takes a rather complicated process for attitude to take effect in practice, which is affected by many factors like culture, tradition, regulation and education. Methods such as Likert-scale have been developed to measure attitude[4]. Thus, it is possible and important to learn about the current KAP status of Chinese people.

The young have always been a group particularly vulnerable to food hazards. They are more prone than other ages to consume food with the risk of safety problems at and outside homes[13]. It is during these formative years that people develop their perceptions toward food safety, as well as toward sources of food safety information.[14]. Education on food safety is helpful for the young because they will need it to develop proper attitude, sound knowledge and skills to understand contemporary food issues[15]. Hence, understanding food safety knowledge, attitude and practices of young students is key to identifying ways to give better education and to minimize the risk of foodborne diseases.

Most food-safety studies have dealt with cooking at home and focused exclusively on adults[16]. Studies aimed at improving food safety in the production process have also been carried out in other establishments like dairy factories, hotels and abattoirs[4,17–19]. In general population, several studies in multiple cities of China showed that public knowledge of and confidence in food safety were poor[20–22]. However, information of KAP on adolescent students was limited because past studies lacked well-designed investigations into this age group on a relatively large sample [23–25]. Therefore, the objective of this study is to understand the food safety-related knowledge, attitude and self-reported practice in a sample of secondary school students in Beijing of China.

## Materials and methods

### Study design and participants

A cross-sectional survey using paper questionnaire was administered on the student body of nine Beijing secondary schools from December 2014 to January 2015. A multi-stage proportionate stratified cluster sampling approach was applied in enrolling participants. First, the 17 districts of Beijing city were stratified into 3 groups: downtown area, urban-rural linking area and rural area, and one district was selected from each group as the target for the study. In each of the three districts one junior high school, one senior high school and one vocational high school were selected from those that had agreed to help and hand out the questionnaires to their students. The sample of districts and schools can be regarded as a convenience sample, which is usually used in exploratory cross-sectional studies[26,27] when conditions do not allow randomization. Through sample size calculation, the number of students needed in each school was determined proportionally based on the Beijing education statistical data (2014–2015 school year). Classes were then randomly selected from the 1^st^ and 2^nd^ grades of each school until reaching the sample size(n = 4200). Once selected, every student in the class was included. Investigators explained the purpose of the study to the students, and informed that the study was completely irrelevant to their school records. A statement on the questionnaire assured the students that all responses would remain anonymous. Questionnaires were completed and collected in the absence of teachers during the class breaks, and were delivered immediately to the researchers. This study was approved by the Ethical Review Board of Peking University Health Science Center (IRB00001052-14013) and the need for participant consent was waived by the ethics committee.

### Procedures

A structured questionnaire was developed prioritizing questions that aligned with the health education guideline for primary and secondary school students[28], as well as the 2011–2015 food safety popularization objectives [29]. The content of the questionnaire was reviewed and modified through expert consultation, which, in addition to demographic questions, addressed food safety knowledge (7 questions), attitudes (3 questions) and self-reported practices (3 Questions)(S1 Appendix).

For knowledge questions, each correct answer was given 1 score, whereas a 0 score was given to wrong answers and the choice of ‘I don't know’, except for questions with multiple correct answers where each wrong answer was given a -1 score[4]. The cut-off points, 80.0%-100.0%, 60.0–79.9% and <60.0% were used to classify the scores of knowledge(varied from -2~15 points) into 3 levels as follows: 1. High level:12–15 scores; 2. Moderate level:9–11 scores; 3. Low level:-2-8 scores[4]. Moreover, the knowledge score was modified (2 scores were added to each score to make it non-negative) to fit with the multivariable regression model used in further analysis.

### Data analysis

Answers in the questionnaires were entered into the software program EpiData Entry (Chinese version 3.1, EpiData Association, Odense, DK, 2008) and transformed into an electronic database. The accuracy of data entry was checked by consistency test after double blind inputting. Missing data of a certain question was omitted in the analysis of that question. Descriptive analyses were performed using SAS software (version 9.2, SAS Institute Inc., Cary, NC, USA, 2009), after data cleaning and coding. Frequency (%) for categorical data and mean with standard deviation (SD) or quartiles for numerical data (dependent on distribution) were used primarily to summarize and describe the data. Differences between means were tested using t-tests, or Kruskal-Wallis and Wilcoxon rank-sum tests if the data followed skewed distribution, with SNK-Q test applied for multiple comparison. Differences between proportions were tested using Pearson’s chi-square test and Fisher’s exact test if necessary. Factors associated with the modified food knowledge score were identified using multivariable Poisson regression. Differences and effect estimates with P< .05 were considered statistically significant.

## Results

### Demographic information

In total, 4220 participants completed the survey questionnaire. The demographic characteristics of the respondents were shown in Table 1. Respondents ranged from 11 to 20 years old. The median age was 14 years, with Q1 of 13 years and Q3 of 16 years. The numbers of boys and girls were almost the same (49.7% to 50.3%). 48.1% of respondents studied in downtown area, 35.8% in urban-rural linking area and 16.1% in rural area. The majority of respondents were junior high school students (51.5%), followed by senior high school students (34.2%), and vocational high school students (14.3%). Only 10% of respondents were boarding students. As much as 61.1% of the respondents’ parents had a tertiary education background. Overall, 4.2% of respondents reported to have smoked in the last month, while 14.8% reported alcohol use in the last month. As expected, age was highly associated with school type(*P*<0.001), thus school type was used instead of age in all further analysis.

**Table 1:** Demographic characteristics of participants and factors associated with the modified food safety knowledge score.

Characteristics	n (%)	Parameter estimates^a^		
		Regression coefficient	P-value	RR (95% CI)^b^
Gender (n = 4220)				
Male	2097(49.7)	-.008	.07	0.992(0.983,1.001)
Female*	2123(50.3)			
Age^c^ (n = 4220)	14(13–16)			
Minimum	11^c^			
Maximum	20^d^			
Region (n = 4220)				
Downtown area	2030(48.1)	.017	.01	1.017(1.004,1.030)
Urban-rural linking area	1511(35.8)	.025	<0.001	1.026(1.012,1.039)
Rural area*	679(16.1)			
School type (n = 4220)				
Junior high school	2173(51.5)	.010	.14	1.010(0.997,1.024)
Senior high school	1442(34.2)	.055	<0.001	1.057(1.042,1.072)
Vocational high school*	605(14.3)			
Educational background of parents (n = 4147)				
Primary/elementary*	500(12.1)			
Secondary/comprehensive	1009(24.3)	.025	.12	1.026(0.994,1.059)
Tertiary/university	2532(61.1)	.040	.009	1.041(1.010,1.072)
Unknown	106(2.5)	.066	.04	0.936(0.878,0.997)
Residence (n = 4203)				
Boarding	420(10.0)	-.019	.02	0.981(0.966,0.997)
Non-boarding*	3783(90.0)			
Academic ranking in class (n = 4210)				
High	1667(39.6)	.033	< .001	1.033(1.021,1.046)
Medium	1629(38.7)	-.003	.65	0.997(0.985,1.009)
Low*	914(21.7)			
Smoking in the last month (n = 4217)				
Yes	4046(95.9)	.084	< .001	1.088(1.058,1.118)
No*	171(4.1)			
Alcohol use in the last month(n = 4211)				
Yes	3587(85.2)	.018	.009	1.018(1.004,1.031)
No*	624(14.8)			

^a^Parameters were estimated from multivariate Poisson regression

^b^Relative risk with 95% confidence interval; ^c^Age was described using median (IQR), minimum and maximum

*Reference group in the regression model.

### Knowledge regarding food safety

**Fig 1:**
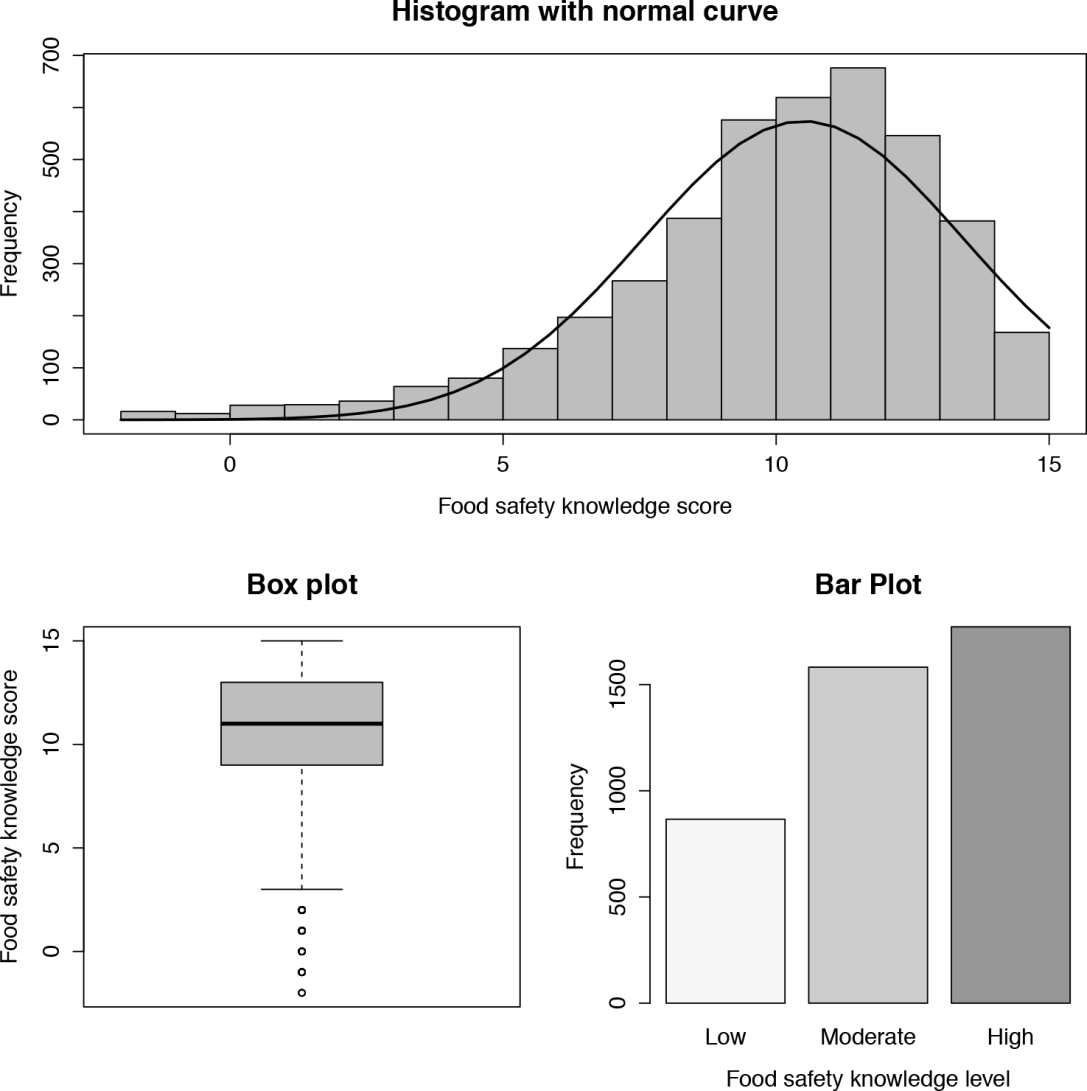
Description of food safety knowledge.

Respondents answered a total of seven questions regarding food safety knowledge, of which some had multiple correct answers (Table 2). The knowledge scores ranged from -2 to 15 and followed right-skewed distribution, with a median of 11(IQR [interquartile range], 9–13). 4.0% of respondents (n = 168) got full score and very few (2.0%, n = 85) scored below 3. The majority of respondents (42.0%) had a high level of knowledge, 37.5% a moderate level and 20.5% a low level (Fig 1).

**Table 2:** Distribution of responses to the knowledge questions regarding food safety.

Questions	Options	Frequency	Percentage(%)
“What’s the usual cause of food poisoning?”**(n = 4213)			
	Bacteria*	2801	66.5
	Chemicals*	2656	63.0
	Toxic animals and plants*	2650	62.9
	I don’t know	302	7.2
“Is food decay caused by microorganisms?”(n = 4195)			
	Yes*	2999	71.5
	No	482	11.5
	I don’t know	714	17.0
“Do you know about the Diet Guideline for Chinese Citizens?” (n = 4214)			
	Yes*	2356	55.9
	No	1858	44.1
“Which of the following do you think belongs to common food categories” **(n = 4209)			
	Cereal*	3228	76.7
	Milk and beans*	3186	75.7
	Meat, fish and eggs*	3476	82.6
	Vegetables and fruits*	3639	86.5
	Oils and fats*	1673	39.7
	I don’t know	157	3.7
“What in the following do you think is healthy lifestyle?” ** (n = 4174)			
	Balanced diet*	4011	96.1
	Take many dietary supplements	361	8.6
	Control body weight moderately*	2989	71.6
	Eat more and sleep more	231	5.5
“Which of the following statement is right regarding food additives?” **(n = 4200)			
	Food additives shouldn’t be used	573	13.6
	Using food additives illegally may cause food safety problems*	3115	74.2
	Using food additives reasonably is good for people’s health and enriches food variety*	2935	69.9
	I don’t know	180	4.3
“Which of the following statement is right regarding food expiration date?” (n = 4200)			
	Expired food can be eaten as long as it appears good	194	4.6
	Expired food can be eaten after heating or boiling	133	3.2
	Expired food can’t be eaten*	3749	89.3
	I don’t know	124	3.0

*Correct response to each question.

** Multiple answers are correct to the question.

Distribution of responses to each knowledge question is shown in Table 2. Among the respondents, 66.5%,63.0%,62.9% knew that bacteria, chemicals, toxic animals and plants were the usual causes of food poisoning, respectively, but only 30.3% chose all the 3 answers at the same time; less than half(39.7%) knew that ‘oils and fats’ was one of the food categories while most was right about the other 4 types of food, with only a quarter choosing all the five right answers at the same time(28.3%); A small percentage incorrectly thought ‘take many dietary supplements’ and ‘eat more and sleep more’ are healthy lifestyle(8.6% and 5.5%, respectively); the majority knew that illegal use of food additives could result in food safety problems (74.2%) and reasonable use of food additives could enrich food variety and benefit people’s health (69.9%), whereas 13.6% incorrectly believed food additives should not be used; a large majority knew that expired food couldn’t be eaten, yet a small number still thought that expired food could be eaten after boiling or heating(3.2%), or as long as it appeared good(4.6%)(Table 2).

### Factors associated with the modified food safety knowledge score

Factors associated with the modified food safety knowledge score from multivariable Poisson regression are also shown in Table 1. All demographic characteristics except age were included in the model. The results showed that all variables but gender were significant after adjusting for the other variables in the model. Specifically, respondents who studied in downtown area and urban-rural linking area had higher scores than those who studied in rural area; senior high school students scored higher than vocational high school students, while differences were not found between junior high school students and vocational high school students; respondents whose parents had received tertiary education performed better than those whose parents had only primary education, whereas no significant difference between the primary and secondary groups; boarding students got lower scores than non-boarders; those who reported to rank higher in academic records were found to have higher scores than those with lower ranking, while no significant difference between medium and lower ranking groups; those who had not smoked in the last month had more scores than those who had, and same was found in alcohol use.

### Attitude and practice towards food safety

Table 3 shows the distribution of respondents’ attitude and practice towards food safety. Respondents’ confidence in food safety situation of China was low. The greatest number of respondents (38.9%) disagreed with ‘The general food safety situation in China is good’, followed by ‘not sure’ (29.4%), ‘agree’ (16.1%), ‘strongly disagree’ (14.7%) and very few chose ‘strongly agree’ (0.9%). A majority of respondents strongly agreed (53.3%) or agreed (42.0%) that they were worried about the food safety of small restaurants and street peddlers. Almost all respondents reported they cared about food safety issue (96.4%, ‘strongly agree’ or ‘agree’) and they averaged significantly higher in knowledge score than those who reported not to care(‘Disagree’), according to bivariate analysis using Wilcoxon rank-sum test (*P*< .001).

**Table 3:** Distribution of the respondents’ attitude and practice towards food safety.

Questions	Options	Frequency	Percentage (%)
Attitude			
“The general food safety situation in China is good.” (n = 4211)			
	Strongly agree	37	0.9
	Agree	680	16.1
	Not sure	1240	29.4
	Disagree	1637	38.9
	Strongly disagree	617	14.7
“I am worried about the food safety of small restaurants and street peddlers.” (n = 4211)			
	Strongly agree	2246	53.3
	Agree	1767	42.0
	Disagree	198	4.7
“I care about food safety issue.”(n = 4214)			
	Strongly agree	2064	49.0
	Agree	2000	47.5
	Disagree	150	3.6
Practice			
“I read information on the food labels when buying them.” (n = 4213)			
	Always	2804	66.6
	Occasionally	1279	30.4
	Never	126	3.0
“I buy food from small restaurants and street peddlers.” (n = 4208)			
	Always	271	6.4
	Occasionally	2651	63.0
	Never	1283	30.5
“Have you ever decided to eat less of certain food because you worry about its safety?” (n = 4205)			
	Yes	1836	43.7
	No	2364	56.3

About reading information on the labels when purchasing food, 66.6% respondents answered always, 30.4% occasionally and 3.0% never. Those who reported reading labels more often had a greater knowledge score based on Kruskal-Wallis test and multiple comparison (*P*< .001). A majority of respondents reported occasionally to have bought food from small restaurants and street peddlers (63.0%), with 6.4% always and 30.5% never. This was significantly associated with their concern about the safety of food prepared by these places according to Pearson Chi-square test (*P*< .001), in that those with more concerns tended to buy less often. More than half of respondents (56.3%) reported they had ever considered taking less or none of the food which they thought to have safety problem.

## Discussion

A large proportion of foodborne diseases are caused by food improperly prepared or mishandled at home, in restaurants or markets[3]. In China, most school-going adolescents do not cook or prepare food themselves[30]. Despite eating food handled by parents or school canteens, some would purchase food from public services such as markets and restaurants, but the safety of food prepared in these places especially by small restaurants and street peddlers has been a great public concern for years. Thus, unlike studies addressing food handling practices[31–34], this study mainly focused on the students’ role as food consumers and on their basic food safety knowledge and practices when buying and storing food.

The findings of this study show that knowledge level was generally good among secondary school students in Beijing, with nearly half ranked at high level. Multivariate analysis showed that all the investigated demographic characteristics had an impact on food safety knowledge, except gender: students studying in more developed area, at more senior grade, with better academic records, having parents with higher education, or without a habit of smoking/drinking tended to have more knowledge on food safety; no significant difference was found between junior high school and vocational high school students; non-boarding students performed better than boarding students. These results suggest that targeted effort should be made to further improve the current food safety knowledge level in secondary school students, with general education playing a very important role in the process. Key subgroups which might be more viable to locate and target for such improvement through education include vocational high school students, boarding students and those studying in less developed regions. A cluster trial has assessed the food safety education targeted on primary school students in remote areas of west China and proved it effective in improving knowledge and behavior at group level[12]. It is important to address that, different from this research, the findings of several similar studies carried out in other Chinese cities (mostly second tier cities)[12,23,25,35] showed a relatively low level of food safety knowledge among students, which is somehow consistent with the influencing factor analysis results in this study because Beijing as the capital is one of the most developed regions of China.

Despite the high knowledge level, there were still several misunderstandings found on a considerable number of students. Firstly, some students incorrectly believed that taking a lot of dietary supplements is good for health. They might have been influenced by the flooding and misleading advertising of these products, especially when there is a lack of accessible proper education.[36]. Secondly, compared with other food sources, oils and fats being an essential element of human body seemed most unrecognized with the students. This might be largely because they heard too much about diseases caused by body fat and unhealthy lifestyles when people consume too much fat in their diet. Some extremists even claim that a fat-free diet is healthy and beneficial. These ideas are wrong and dangerous as fat is an integral part of human diet. Thirdly, in China there are a lot of food with “No Preservatives” on its label to convince consumers that they are natural and healthy, but this is misleading because lacking reasonable use of preservatives is exactly why many food safety problems occur[37,38]. The false advertising led to an incorrect impression for some students that food additives are harmful and should not be used. In fact, food additives are safe as long as used in accordance with legal standards, and 99.6% of the food sampled by Chinese authority used qualified and proper amount of food additives in 2015[11]. It cannot be ignored, though, that there still are cases where additives are abused especially in some small restaurants and food processors, resulting in food safety problems[39]. That is why the correct information about additives should be communicated to students in order that they do not fear their use nor lose alert on their abuse. In a word, to address such misunderstandings, more systematic and targeted education is much needed among students.

We found that confidence in food safety was low among Beijing secondary school students, with more than half thought the general food safety situation was not good in China. The result was consistent with similar studies focusing on Chinese adults[20,21]. Maybe this is because Chinese people’s sentiment has been hurt by quite a few poisonous food events reported in the past years, such as ‘melamine milk’, ‘gutter oil’ and pork products contaminated with clenbuterol[9]. These events have shocked the public and their aftermath can last for a long time, affecting the public judgment on current general food safety of China. Expectedly, great attention was paid to food safety issue by most students, which was in line with their high knowledge level. This, together with relatively high knowledge level and low confidence showed their worry and anxiety in this issue. Governments should be responsible for ensuring consumer’s trust in authorities and confidence in the safe food supply[3], which is urgently the case in China. Chinese government has made food safety a public health priority. Policies and regulatory frameworks to manage food safety risks have been developed to build and implement a food safety system which ensures that food producers and suppliers along the whole food chain operate responsibly and supply safe food to consumers[40,41]. Meanwhile, many ways of education have been actively adopted by the government not only to spread knowledge but also to show the public what effort is being made by it. Since 2011, the thematic event of “China Food Safety Publicity Week” has been held every June aimed at improving awareness and confidence of food safety and safety regulations for the public. With these measures taken, future studies might discover some improvement in confidence.

With respect to food safety practice, the students showed a good habit of reading labels when buying packaged foods. Buying food from small restaurants and street peddlers was very common, despite as many as 95.3% of the students had concern about the safety of food prepared by them. Though association was found that students with more concern bought less, it is still easy to see from the data that many chose to omit their concerns and continued buying food from these places. In fact, more than 40% reported not to have reduced consumption of such food because of safety concern. This, according to the stages of change model[42], suggests that generally a number of students were in the stage of contemplation and preparation (some problems recognition, but ambivalence about the need to change). Strategies and education increasing self-efficacy about food safety may be a way to help students move to the next stage of action (concrete changes in behavior are occurring).

There are a few limitations of this study. First, the districts and schools were not randomly selected, which may limit the generalizability of results in this study to all secondary school students in Beijing. This was because not every school was willing to participate in the research. Second, scales were not applied to assess attitude and practice, which should be improved in further studies. Third, as is usually found in other researches of self-reported practices, respondents may typically provide favorably biased results of desired behaviors[43], even though efforts have been made in this study to minimize this potential by telling respondents that their answers are anonymous and not related to school records. Additionally, the handling of missing data of each individual question, though not much missed compared to the sample size, does have the potential to bias the results. Finally, this study cannot provide any information about causal relationships because of its cross-sectional nature.

In conclusion, based on the results of this study, secondary school students should be a vital target group for food safety education because there is still much space for improvement in their knowledge, confidence is low while many still do not bother to take action and they are easy to be influenced by misleading information. Given that boarding students, vocational high school students and those in less developed regions have fewer knowledge scores, these subgroups might particularly benefit from education. The government plays an important role in food safety education and publicity for students, so do social media, schools and parents. Therefore, advocated by the government, more structured and targeted education should be routinely carried out through class teaching as well as through mass media. It is also recommended that further studies make more efforts to evaluate the effectiveness of such interventions.

## Supporting information

S1 Dataset Anonymized raw data of the survey. (XLS)

S1 Appendix Questionnaire used in the survey in both Chinese and English. (PDF)
