# Supplementary material for: Food safety knowledge, attitude and self-reported practice of secondary school students in Beijing, China: A cross-sectional study
Source: PLoS One. 2017 Nov 2;12(11):e0187208. doi: 10.1371/journal.pone.0187208 (PMC5667794; doi:10.1371/journal.pone.0187208)
Supplement: S1 Appendix — (PDF) [file pone.0187208.s002.pdf]

编号：❑❑❑❑❑❑❑❑

《北京市中学生食品安全认知调查问卷》

同学们，你们好！

你们正处于生理、心理迅速发育的时期，也是世界观、价值观形成的关键阶段。食品安全是关系国计民生的大事，对你们的健康成长也有着很大的影响。本次调查希望了解你们对于食品安全问题的知晓情况，以便于制定帮助你们获得正确知识、保护你们健康的政策和措施。为了你和家人、朋友的健康，请配合我们做好这项调查（大概需要 10-15 分钟）。问题没有对错之分，根据你的真实情况回答即可。**我们保证，所有你提供的信息都是保密的，仅用于科学研究。**谢谢配合！

请注意：

1．不必在问卷上写自己的名字。

2．答案与成绩无关，同学间不必商量。

3．不必费时斟酌，你的最初反应是最真实的。

4．**你的回答是保密的，老师和家长绝不会知道你的答案，请根据自己的真实体验放心解答。**

5．回答时在对应选项下画“√”即可；如无说明“可多选”，答案只有一个。

第一部分

A01.性别：①男　②女

A02.出生日期：______年_____月_____日

A03.民族：______　①汉族　②其他，请注明_______

A04.你父母的文化程度是：父亲____　母亲____

①小学及以下 ②初中 ③高中/技校/中专 ④大专/大学本科 ⑤研究生及以上 ⑥不知道

A05.本学期，你是否为住宿生？

①是 ②否

A06.过去 12 个月里，与同班同学相比，你认为自己的学习成绩如何？

①前 10% ②前 10%~前 30% ③中等 ④后 10%~后 30% ⑤后 10%

A07.你最近一个月吸过烟吗?

①没有 ②平均每天不到 1 支 ③平均每天 1-3 支 ④平均每天 3-10 支 ⑤平均每天 10 支以上

A08.你最近一个月饮过酒吗?

①没有 ②1-3 次 ③4-6 次 ④6 次以上

第二部分

B01.食物中毒有哪些常见原因？（可多选）

①细菌引起 ②化学物质引起 ③有毒动植物引起 ④不知道

B02.食物腐败变质是由微生物引起的，这种说法对吗？

①对 ②不对 ③不知道

B03.你听说过中国居民膳食指南吗？

①听说过 ②没听说过

B04.食物的常见分类包括以下哪些？（可多选）

①谷类 ②奶类和豆类 ③鱼、禽、肉、蛋类 ④蔬菜和水果类 ⑤油脂类 ⑥不知道

B05.你认为健康的生活方式包括以下哪些内容（可多选）：

①平衡膳食　②多吃保健品、营养品　③适度控制体重　④多吃多睡

B06.以下关于食品添加剂的说法正确的有哪些（可多选）：

①食品中不该使用添加剂　②非法使用食品添加剂会带来食品安全问题

③合理使用食品添加剂，对丰富食品生产和促进人体健康都有好处　④不知道

B07.以下关于食品保质期的几种说法，你认为正确的是：

①过了保质期的食品只要看起来没坏还可以吃　②过了保质期的食品加热蒸煮后还可以吃

③不吃超过保质期的食品　④不知道

B08.“我国当前食品安全状况良好”，你同意这个说法吗？

①非常同意　②比较同意　③说不好　④不太同意　⑤非常不同意

B09.你是否担心过小店或者流动摊位卖的食品不卫生？

①经常　②偶尔　③从不

B10.你在乎食品安全问题吗？

①非常在乎　②比较在乎　③不在乎

B11.在购买食品时，你是否会关心食品的生产日期、保质期、质量合格标志等内容？

①总是关心　②偶尔关心　③不关心

B12.你在学校周边的小店或流动摊位购买“小食品”吗？

①经常　②偶尔　③从不

B13.你是否曾因为担心特定的食品安全问题而减少了相应食品的摄入？

①是　②否

问卷到此结束，多谢合作！

（此栏由调查员填写）　调查员签名：____________　调查日期：___________年

月_____日

No : ❑❑❑❑❑❑❑

Questionnaire of the Beijing secondary school students' food safety KAP survey

Dear respondents,

Food safety is a very important issue worldwide and has major implications on everyone. In this survey, we would like to ask you some questions regarding your knowledge, attitude and practice toward food safety in order to provide suggestions to better protect you and your friends from food poisoning. It will take you about 10 to 15 minutes to finish the questionnaire. We assure you that all the information you provide is confidential and will only be used for research purpose. Thank you for your cooperation！

Please notice:

1. You don't need to write your name on the questionnaire.
2. Your responses have nothing to do with your academic records. Please don't discuss with each other.
3. Please answer at your fist response.
4. Teachers and parents will not know your responses.
5. Please put a "√" on the number your choice. There is only one correct answer unless otherwise marked.

Part 1

A01.Gender：①Male ②Female

A02.Birthday：____Y_____M____D

A03.Race：①Han ②Others, Please specify_________

A04.Educational background of your parents：Father_____ Mother_____

①Primary school or under ②Junior high school ③Senior high school or vocational high school ④Bachelor's degree or diploma ⑤Master's degree or above ⑥I don't know

A05.Do you live on campus this year?

①Yes ②No

A06.How is your academic ranking in class during the last 12 months?

①Top 10% ②Top 10% to 30% ③Medium ④Last 10%~30% ⑤Last 10%

A07.Have you ever smoked in the last month?

①No ②Less than 1 cigarette per day ③1-3 cigarettes per day ④4-10 cigarettes per day ⑤More than 10 cigarettes per day

A08.Have you ever drunk alcohol in the last month?

①No ②1-3 times ③4-6 times ④More than 6 times

Part 2

B01.What's the usual cause of food poisoning? (multiple correct answers)

①Bacteria ②Chemicals ③Toxic animals and plants ④I don't know

B02.Is food decay caused by microorganisms?

①Yes ②No ③I don't know

B03.Do you know about the Diet Guideline for Chinese Citizens?

①Yes ②No

B04.Which of the following do you think belongs to common food categories? (multiple correct answers)

①Cereal ②Milk and beans ③Meat, fish and eggs ④Vegetables and fruits ⑤Oils and fats ⑥I don't know

B05.What in the following do you think is healthy lifestyle? (multiple correct answers)

①Balanced diet ②Take many dietary supplements ③Control body weight moderately ④Eat more and sleep more

B06. Which of the following statement is right regarding food additives? (multiple correct answers)

①Food additives shouldn't be used ②Using food additives illegally may cause

food safety problems ③Using food additives reasonably is good for people's health and enriches food variety ④I don't know

B07. Which of the following statement is right regarding food expiration date?

①Expired food can be eaten as long as it appears good ②Expired food can be eaten after heating or boiling ③Expired food can't be eaten ④I don't know

B08. Do you agree that the general food safety situation in China is good?

①Strongly agree ②Agree ③Not sure ④Disagree ⑤Strongly disagree

B09. Do you agree to be worried about the food safety of small restaurants and street peddlers?

①Strongly agree ②Agree ③Disagree

B10. Do you agree that you care about food safety issue?

①Strongly agree ②Agree ③Disagree

B11. Do you read information on the food labels when buying them?

①Always ②Occasionally ③Never

B12. Do you buy food from small restaurants and street peddlers?

①Always ②Occasionally ③Never

B13. Have you ever decided to eat less of certain food because you worry about its safety?

①Yes ②No

Questionnaire is completed. Thank you for your cooperation!

Signature of investigator ：___________ Date of Survey ：_______Y_____M_____D
